# Organoporosity Evaluation of Shale: A Case Study of the Lower Silurian Longmaxi Shale in Southeast Chongqing, China

**DOI:** 10.1155/2014/893520

**Published:** 2014-08-11

**Authors:** Fangwen Chen, Shuangfang Lu, Xue Ding

**Affiliations:** Institute of Unconventional Hydrocarbon and New Energy Sources, China University of Petroleum (East China), Qingdao 266580, China

## Abstract

The organopores play an important role in determining total volume of hydrocarbons in shale gas reservoir. The Lower Silurian Longmaxi Shale in southeast Chongqing was selected as a case to confirm the contribution of organopores (microscale and nanoscale pores within organic matters in shale) formed by hydrocarbon generation to total volume of hydrocarbons in shale gas reservoir. Using the material balance principle combined with chemical kinetics methods, an evaluation model of organoporosity for shale gas reservoirs was established. The results indicate that there are four important model parameters to consider when evaluating organoporosity in shale: the original organic carbon (*w*(TOC_0_)), the original hydrogen index (*I*_H0_), the transformation ratio of generated hydrocarbon (*F*(*R*_*o*_)), and the organopore correction coefficient (*C*). The organoporosity of the Lower Silurian Longmaxi Shale in the Py1 well is from 0.20 to 2.76%, and the average value is 1.25%. The organoporosity variation trends and the residual organic carbon of Longmaxi Shale are consistent in section. The residual organic carbon is indicative of the relative levels of organoporosity, while the samples are in the same shale reservoirs with similar buried depths.

## 1. Introduction

Petrological characteristics (such as brittle mineral content), micro-nanoscale porosity, total organic carbon, and organic matter maturity of shale play important roles in the accumulation and exploration of shale gas. The nanometre pores in shale are mostly organopores (pores within organic matters in shale) created from hydrocarbon generation during formation subsidence and thermal evolution [1–3]. Many scholars studying the organoporosity of shale gas reservoirs believe that the organopores in organic matters generated during hydrocarbon generation make a significant contribution to gas reservoir space [4–7]. For example, in the Mowry Shale of the Powder River Basin, the organic matter type of the source rocks is type II. If the original organic carbon *w*(TOC_0_) is 6% and the vitrinite reflectance (*R*_*o*_) goes up to 1.2%, the organoporosity is approximately 5% [4, 5]. The surface area of the organopores that approach nanoscale becomes significant and strongly affects the amount of gas stored as the free component versus the adsorbed component [8–10]. Typical methods, such as mercury injection capillary pressure (MICP) measurement and nuclear magnetic resonance (NMR) spectroscopy, can not measure organopores in shale effectively. These macroscopic methods do not involve the direct measurement of individual pores, which range in size for shale gas from a few to tens of nanometres [11, 12]. At present, the methods for measuring porosity of shale are low-temperature N_2_ and CO_2_ gas isotherm adsorption-desorption, scanning electron microscopy (SEM), 3D high-resolution imagery, and total porosity determined by bulk density coupled with skeletal density. Isotherm adsorption-desorption measures the connected pores, which range in size from 0.38 to 100 nanometres in shale, and the measured results are smaller than the actual pore values [13]. Scanning electron microscopy can image the microstructure of shale pores, especially the organopores [14–18], but the observations are limited to 2D sections and cannot be used to perform quantitative evaluation. The difference between skeletal density and bulk density measured by He pycnometry and Hg immersion, respectively, is used to calculate the total porosity of shale [19, 20]. Because of the adsorption of organic-rich shale, inconsistent crushing and sample handling procedures, the difference of relative humidity of the measurement environment, and the solvent extraction pretreatment that may potentially remove some of the solid organic matter, the measured volume has certain errors and defects [20]. Three-dimensional high-resolution imagery can be used to investigate shale microstructure and analyse the characteristics of pores and the porosity of microscale shale cubes [8, 9]. However, smaller sample diameters correspond to higher 3D-reconstruction resolutions [21]. The particle size of quantitatively evaluated shale is 1 to 5 *μ*m in 3D-reconstruction [22]. There is strong heterogeneity in shale [23]. Therefore, smaller samples are less representative. What is more, these methods can not evaluate the organoporosity effectively.

Given that organopores play an important role in determining shale gas reservoir space, it is necessary to study organoporosity. Therefore, taking the Lower Siluriran Longmaxi Shale in the southeast Chongqing as a case study, a method was established for estimating organoporosity, which is useful in the simultaneous exploration and development of shale gas.

## 2. Geological Setting

**Figure 1:**
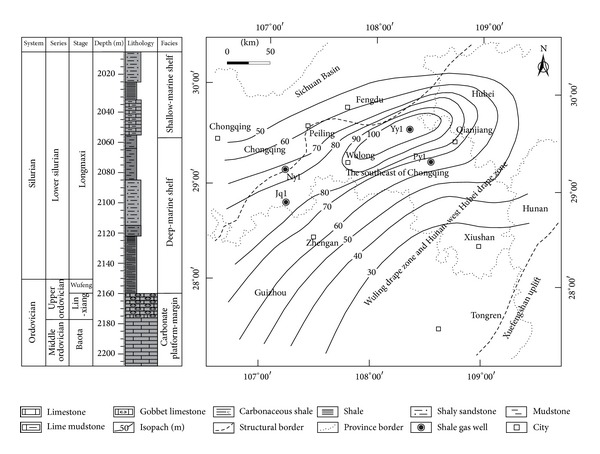
Stratigraphic column of Py1 well and isopach of Longmaxi Shale in southeast Chongqing.

The study area is in southeast Chongqing, China, and covers an area of approximately 1.98 × 10^4^ km^2^. The north, south, and east of it are Hubei Province, Guizhou Province, and Hunan Province, respectively. It belongs to the Yangtze tectonic plate [24, 25] and is located in the Wuling Drape Zone and Hunan-west Hubei Drape Zone. The Xuefengshan Uplift and Sichuan Basin are in the east and northwest of southern Chongqing, respectively (Figure 1).

The residual strata of Paleozoic are Cambrian, Ordovician, and Silurian, and the other layers are denuded or missing. The Lower Paleozoic marine shale mainly including the Lower Cambrian Niutitang Shale and the Lower Silurian Longmaxi Shale is widely deposited in southeast Chongqing. They have the characteristics of large thickness, deep buried depth, and well-developed fractures [26–28]. In the Longmaxi formation, the lower and middle parts belonged to deep-marine shelf sedimentary environment and the upper part to shallow-marine shelf sedimentary environment. Grey and black carbonaceous shale, shale, and grey shaly sandstone are in the lower part, grey and black mudstone is found in the middle part, grey lime mudstone, and shale and shaly sandstone are present in the upper part. Grey and black carbonaceous shale and shale in the lower part of Longmaxi formation with a range of approximately 30–100 metres called Longmaxi Shale are the target strata of shale gas exploration and development (Figure 1). In the study area, the thickness of Longmaxi shale in Py1 well is about 70 metres with a total organic carbon *w*(TOC) range of 0.27–4.25% and average value is 1.90%. The organic matter are types II_1_-I [29], with a vitrinite reflectance (*R*_*o*_) range of 1.90–3.09%, and average value is 2.62%, which are calculated by bitumen reflectance [30].

## 3. Model Creation

Organopores are within organic matters in shale, due to hydrocarbon generation during formation subsidence and thermal evolution. These pores are important for storage and perhaps transfer of gas molecules through shale [23]. The diameter of these organopores is generally less than 1 *μ*m. Thus, they are considered nanometre pores. The sizes of nanometre pores in organic matter mainly range from 80 to 100 nm [17, 22]. For example, in Barnett Shale, a single 10.8-*μ*m-diameter organic matter contained more than 1,000 nanopores of various shapes and sizes [1]. The diameters of intragranular nanopores do not appear to be directly related to the size of the organic matters. Both large and small nanopores can be found in organic matters of all sizes. The total volume of the organopores is mainly related to the total organic carbon level, hydrogen index, maturity, and diagenesis (compaction effect). The paper will discuss how to evaluate the organoporosity of shale.

According to the material balance principle, under ideal conditions, the total volume of organopores is the volume of organic matter consumed by generating hydrocarbon (Formula (1)) [5]:

(1) $$\begin{array}{c} {Ф}_{\text{organic}}^{\prime }=w\left({\text{TOC}}_{0}\right)\cdot I_{\text{H}0}\cdot F\left(R_{o}\right)\cdot \frac{\rho_{\text{rock}}/\rho_{\text{kerogen}}}{1000}, \end{array}$$

where *Ф*_organic_′ is the organoporosity of shale under ideal conditions (%), *w*(TOC_0_) is the weight percent of the original total organic carbon (%), *I*_H0_ is the original cracking hydrocarbon of unit quality organic carbon (mg/g), *F*(*R*_*o*_) is the transformation ratio of oil and gas generated from organic matter (%), which is correlated with maturity, *ρ*_rock_ is the density of shale (g/cm^3^), and *ρ*_kerogen_ is the density of kerogen, which is approximately 1.2 g/cm^3^ [16, 31]. The term *w*(TOC_0_) · *I*_H0_ represents the weight of convertible organic matter. Then the term *w*(TOC_0_) · *I*_H0_ · *F*(*R*_*o*_) is the weight of organic matter consumed by generating hydrocarbon in thermal evolution. The term *ρ*_rock_/*ρ*_kerogen_ is simply a density conversion from weight percent to volume percent. The last term 1000 is a unit conversion from mg to g.

Because of compaction and bituminization process in actual geological conditions, the organoporosity calculated under ideal conditions should be corrected. On the basis of Formula (1), a model was established for evaluating the organoporosity in shale as follows (Formula (2)):

(2) $$\begin{array}{c} {Ф}_{\text{organic}}^{}=w\left({\text{TOC}}_{0}\right)\cdot I_{\text{H}0}\cdot F\left(R_{o}\right)\cdot \frac{\rho_{\text{rock}}/\rho_{\text{kerogen}}}{1000}\cdot C, \end{array}$$

where *Ф*_organic_ is the organoporosity of shale in actual geological conditions (%) and *C* is the organopore correction coefficient.

In the model, there are four important parameters: the original total organic carbon (*w*(TOC_0_)), the original hydrogen index (*I*_H0_), the transformation ratio of oil and gas generated from organic matter (*F*(*R*_*o*_)), and the organopore correction coefficient (*C*). This paper will evaluate these parameters in the following part.

## 4. Model Parameter Evaluation

### 4.1. Transformation Ratio of Hydrocarbon Generated from Organic Matter

To calculate the transformation ratio of hydrocarbon generated from organic matter in Longmaxi Shale of southeast Chongqing, the Xiamaling Shale in the Huabei Platform and crude oil sample generated by the Cambrian source rocks in Tarim Basin were selected for pyrolysis experiments because the shale is similar to the source rocks of the Longmaxi formation and has a relatively lower maturity. Xiamaling Shale is type II_1_ with a vitrinite reflectance (*R*_*o*_) of approximately 0.5%, and the *w*(TOC) is 5.98% (Table 1). The maturity of Longmaxi Shale is relatively high with average vitrinite reflectance 2.62% in study area, and generated oil migrated to overlying strata was suffered bituminization effect. There is no proper crude oil generated from Longmaxi Shale in Sichuan Basin, leaving nature gas and asphaltite. So this paper selected the crude oil sample from the Silurian oil reservoir in the Tarim Basin (Table 2), which is generated by the Cambrian source rocks [32]. The source rocks of Cambrian and Silurian are both marine sedimentary of Paleozoic. The crude oil generated from them may have a certain degree of similarity.

**Figure 2:**
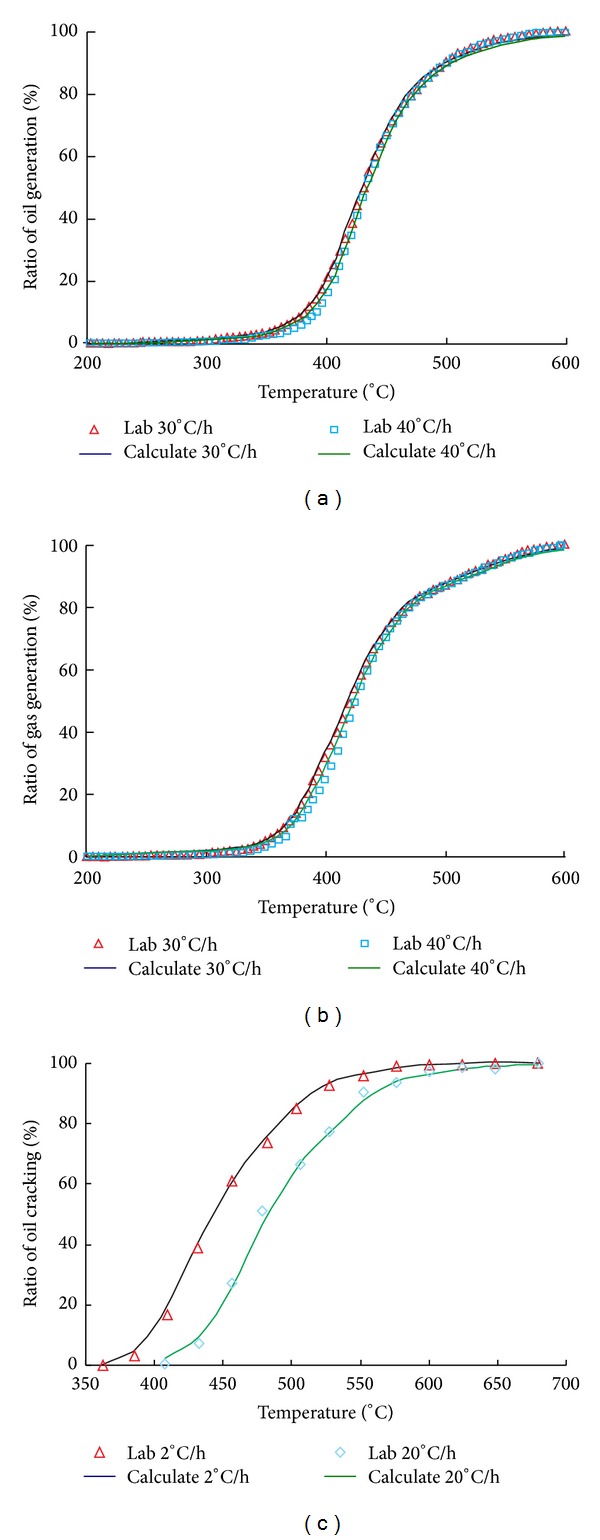
The transformation ratio of oil and gas generated from organic matter at different temperatures and heating rates. (a) The transformation ratio of oil generated from kerogen of Xiamaling Shale. (b) The transformation ratio of gas generated from kerogen of Xiamaling Shale. (c) The transformation ratio of gas generated from oil cracking.

Pyrolysis experiments were performed from 200°C to 600°C with different heating rates using a Rock-Eval-II type pyrolysis instrument (e.g., 30°C/h and 40°C/h). The relationship between the production of hydrocarbons and temperature (or time) was recorded. Next, the transformation ratio of oil and gas at different temperature points was obtained, and the chemical kinetics parameters were calibrated (Figure 2). Further reference studies can be consulted to understand the calibration method [33–35].

**Figure 3:**
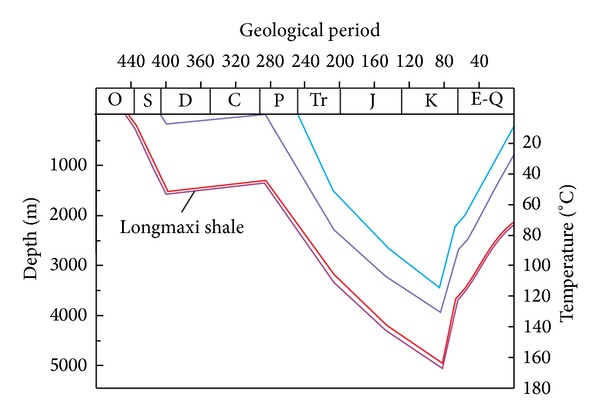
The burial and thermal history of the Py1 well area in southeast Chongqing.

**Figure 4:**
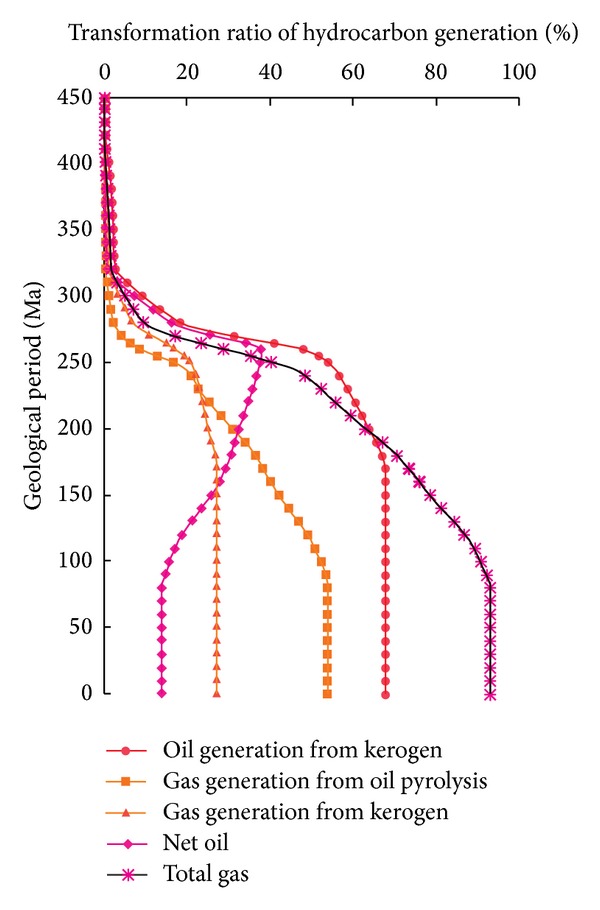
The transformation ratio of hydrocarbon generation with geological period from Py1 well Longmaxi Shale in southeast Chongqing.

On the basis of previous research [36–38], the burial history and thermal history of the study area were analyzed. In Py1 well area, Longmaxi Shale experienced multiple stages of tectonic activity in the southeast of Chongqing. It mainly underwent two rapid subsidence (approximately 450–400 Ma and 285–85 Ma), one slow uplift (approximately 400–285 Ma), and one rapid uplift (85 Ma to now). The maximum buried depth of the shale was approximately 4900 m, and the highest temperature was approximately 160°C (Figure 3). Based on the burial and thermal history, calibration of the chemical kinetic parameters and clarification of the relationship between the transformation ratio of hydrocarbon generated from organic matter (including the transformation ratio of oil and gas generated from kerogen and the transformation ratio of gas generated from oil cracking) and geological period (Figure 4) were combined for the model. In Py1 well area of southeast Chongqing, the main oil and gas generating periods of Longmaxi Shale are 275–190 Ma (Middle Permian to Late Triassic) and 260–80 Ma (Late Permian to Middle Cretaceous).

### 4.2. Original Hydrogen Index and Original Total Organic Carbon

Based on the restoration method of original hydrogen index and original total organic carbon [39] and using the transformation ratio of hydrocarbon generation in combination with the burial and thermal history of the study area (Figure 3), the original hydrogen index value and original total organic carbon of Longmaxi Shale from Py1 well were calculated.

The original hydrogen index value was calculated using the following formula (Formula (3)) [33]:

(3) $$\begin{array}{c} I_{\text{H}0}=I_{\text{H}}+\left(I_{\text{H}0}\cdot F_{o}+B_{0}-B\right)+I_{\text{H}0}\cdot F_{g}, \end{array}$$

where *I*_H0_ is the original hydrogen index value (mg/g), *I*_H_ is the residual hydrogen index value (mg/g), *F*_*o*_ is the transformation ratio of oil generation (%), *B*_*o*_ is the content of native asphalt in source rocks (non-thermally decomposed from kerogen, mg), *B* is the residue oil content calculated from chloroform “A” or the hydrocarbon index by light and heavy hydrocarbon compensation, respectively (mg), and *F*_*g*_ is the transformation ratio of gas generation (%).

The original total organic carbon was calculated using the following formula (Formula (4)) [33]:

(4) $$\begin{array}{c} w\left({\text{TOC}}_{0}\right)=w\left(\text{TOC}\right)\left(1+\Delta I_{\text{H}}\cdot \frac{K}{1000}\right), \end{array}$$

where *w*(TOC) is the weight percentage of the residual total organic carbon (%), Δ*I*_H_ is the recovery content of the hydrogen index (mg/g), and *K* is the coefficient of organic matter conversion into organic carbon, which is approximately 0.85 [5].

**Figure 5:**
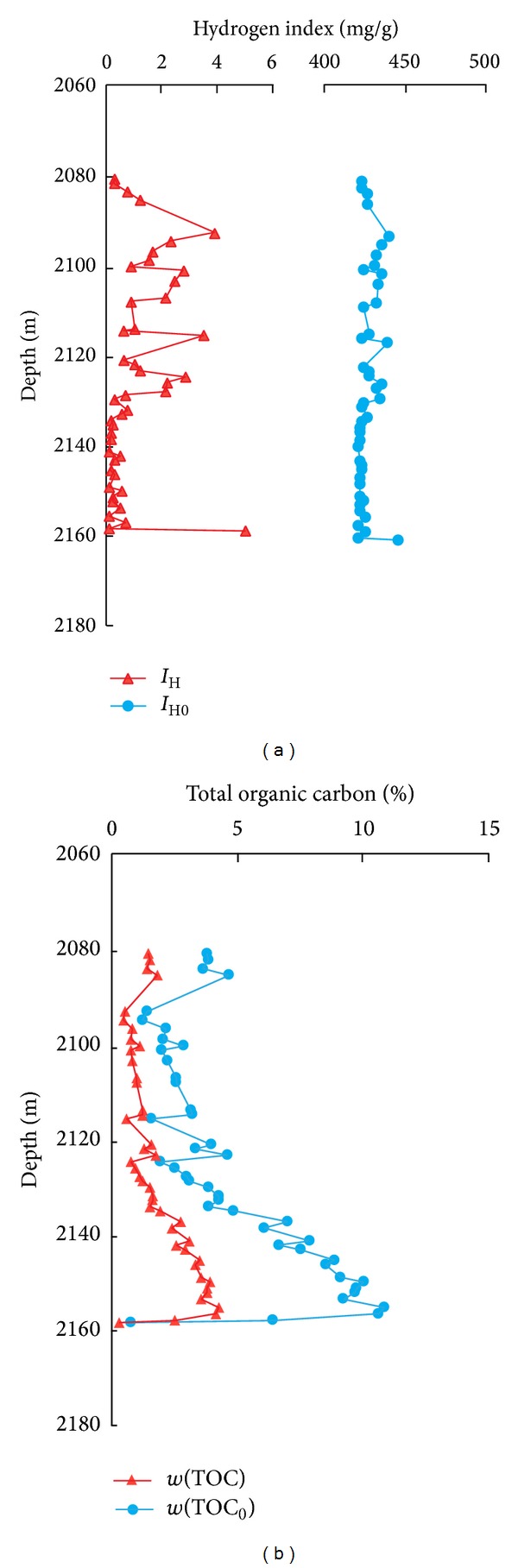
Hydrogen index and total organic carbon values of the Py1 well from Longmaxi Shale in southeast Chongqing. (a) Hydrogen index. (b) Total organic carbon.

The original and residual hydrogen indexes of Longmaxi Shale from Py1 well are in range of 421.31–446.03 mg/g and 0.02–4.93 mg/g, with average values 427.12 mg/g and 1.01 mg/g. The ranges of original and residual total organic carbon are 0.74–10.86% and 0.27–4.25% with average values 4.90% and 1.90%. The original organic carbon is significantly larger than residual organic carbon, but their variation trends with depth are consistent (Figure 5).

### 4.3. Organopore Correction Coefficient

The organopore correction coefficient is the ratio of the volume of actual organopores to pore space, which is formed from hydrocarbon generation under ideal conditions without compaction and bituminization effects in organic matters. In the same set of shale with similar buried depth, because of the environment of temperature and pressure without difference, the process of hydrocarbon generation and expulsion and diagenesis is nearly homogeneous. So the organopore correction coefficients of them are the same. In order to obtain the organopore correction coefficient, the focused ion beam (FIB) milling and scanning electron microscopy (SEM) imaging were used on the shale samples at the same depth. The average organopore surface porosity (SP_organic_) was obtained using the total area of organopores divided by the area of organic matters in several images with the same depth. Organoporosity (*Ф*_organic_) was calculated through combining with the total organic carbon, kerogen density, and bulk density of the samples using Formula (5). Then, using organoporosity (*Ф*_organic_) ratio to organoporosity (*Ф*_organic_′) under ideal conditions, the organopore correction coefficient can be obtained (Formula (1)).

Consider

(5) $$\begin{array}{c} {Ф}_{\text{organic}}={\text{SP}}_{\text{organic}}\cdot w\left(\text{TOC}\right)\cdot \frac{\rho_{\text{rock}}}{\rho_{\text{kerogen}}}\times 100\%, \end{array}$$

where *Ф*_organic_ is the organoporosity of shale in actual conditions (%) and SP_organic_ is the average organopore surface porosity in organic matters.

**Figure 6:**
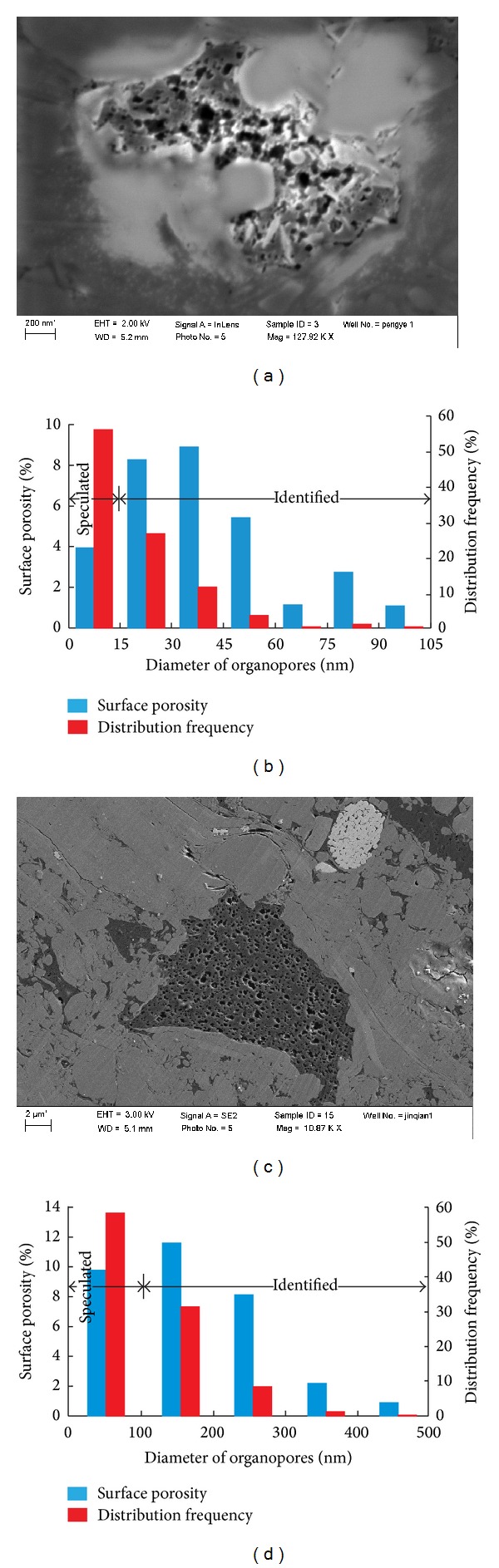
The organic surface porosity and distribution frequency of the Py1 well from Longmaxi Shale in southeast Chongqing.

Using the focused ion beam (FIB) milling and scanning electron microscopy (SEM) imaging on the samples of Py1 well Longmaxi Shale, the surface porosity of organopores was recognized and analyzed by photo identification and forecast in two SEM images, which image two different organic matters in the same sample (Figures 6(a) and 6(c)). The depth of the sample is 2079.99 meters from Py1 well with the TOC value 1.45% and the rock density value 2.65 g/cm^3^. With the increasing of SEM imaging magnification, photo resolution is increased, and the smaller organopore size can be identified. The organopores smaller than the resolution of SEM were unable to be identified. The organopore surface porosity (SP_organic_) is composed of the identified and unidentified organopores in SEM images. The contribution of identified organopores to SP_organic_ can be easily counted. For the unidentified organopores, the contribution of them to SP_organic_ was calculated through the following steps. (1) The distribution frequency of identified organopores in different sizes was analyzed through SEM images. (2) According to the distribution frequency of identified organopores in different sizes, the distribution frequency and size of unidentified organopores were speculated. (3) Assuming that the unidentified organopores are approximate spheres, the contribution of unidentified organopores to SP_organic_ can be calculated using the area formula of a circle.

Combined with the SP_organic_ of identified and unidentified organopores, the surface porosities of different diameter organopores were obtained (Figures 6(b) and 6(d)). The column diagrams of Figures 6(b) and 6(d) are, respectively, corresponding to SEM images of Figures 6(a) and 6(c). The organopore surface porosities of the two SEM images are 32.30% and 28.07% and their average value is 30.19%. The organoporosity value is 0.97% according to Formula (5), while the organoporosity value is 3.20% using the material balance principle (Formula (1)) at the same depth of Py1 well. Finally, the organopore correction coefficient was calculated using the organoporosity value 0.97% in actual geological conditions (*Ф*_organic_) divided by the organoporosity value 3.20% under ideal conditions (*Ф*_organic_′) according to Formula (1) and Formula (2). The organopore correction coefficient is 0.303.

## 5. Organoporosity of Shale

**Figure 7:**
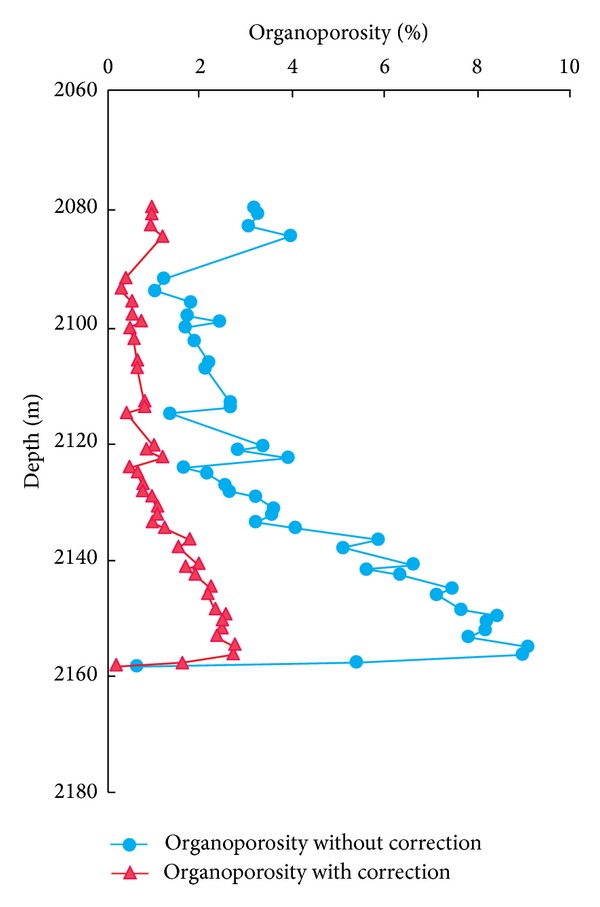
The organic porosities of the Py1 well from Longmaxi Shale in southeast Chongqing.

Using the transformation ratio of hydrocarbon generated from organic matter, the original hydrogen index, the original total organic carbon, and the organopore correction coefficient of the Py1 well Longmaxi Shale in southeast Chongqing (Figures 4, 5, and 6), the organoporosity was calculated. The organoporosity without correction range is 0.66–9.10%, and the average value is 4.14%, whereas the organoporosity with correction range is 0.20–2.76%, and the average value is 1.25%. The organic porosities of Py1 well Longmaxi Shale before and after correction were decreased gradually from bottom to top, and both have the same variation trend with depth (Figure 7). And this variation trend and the residual organic carbon are consistent. So the residual organic carbon can indicate the relative levels of organoporosity, while the samples are in the same shale reservoirs with similar buried depths.

## 6. Conclusions

(1) Using the material balance principle and chemical kinetics, an organoporosity evaluation modelling method for shale rocks was developed. The important parameters for the method are the transformation ratio of hydrocarbon generated from organic matter, the original hydrogen index, the original total organic carbon, and the organopore correction coefficient.

(2) The organoporosity of the Lower Silurian Longmaxi Shale in the Py1 well is from 0.20 to 2.76%, and the average value is 1.25%.

(3) The organoporosity variation trends and the residual organic carbon of Longmaxi Shale are consistent in section. The residual organic carbon is indicative of the relative levels of organoporosity, while the samples are in the same shale reservoirs with similar buried depths.

## Figures and Tables

**Table 1:** Geochemical characteristics of the shale samples.

Samples	Organic matter type	Vitrinite reflectance *R*_*o*_, (%)	Total organic carbon *w*(TOC_0_), (%)	Max temperature *T*_max⁡_, (°C)	Free hydrocarbon *S*_1_, (mg/g)	Cracking hydrocarbon *S*_2_, (mg/g)	Hydrogen Index *I*_H_, (mg/g)	Sedimentary period
Xiamaling Shale	II_1_	0.5	5.98	439	0.45	19.13	320	Precambrian
Longmaxi Shale	II_1_-I	2.62	1.90	455	0.01	0.31	1.01	Lower Silurian

**Table 2:** Characteristics of the crude oil sample from Cambrian source rocks in Tz62 well.

Source rock	Reservoir	Sampling depth (m)	Density g/cm^3^	Saturated (%)	Aromatic (%)	Nonhydrocarbon and asphaltene (%)
Cambrian	Silurian	4052.88~4073.58	0.931	53.256	33.285	13.459
